# Developing, conducting and evaluating the internship preparatory program (Ipp)

**DOI:** 10.1016/j.amsu.2021.103215

**Published:** 2022-01-01

**Authors:** Abeer S. Al Shahrani, Samah F. Ibrahim, Norah M. AlZamil, Eman S. Soliman, Lamya A. Almusharraf, Amel A. Fayed, Noreen Mirza

**Affiliations:** a Department of Clinical Sciences, College of Medicine, Princess Nourah Bint Abdulrahman University, P.O.Box 84428, Riyadh 11671, Saudi Arabia; b Faculty of Medicine, Zagazig University, Egypt; c College of Physcians and Surgeons, Karachi, Pakistan

**Keywords:** Internship, Saudi-Med, Soft skills, Surgical skills, Medical, Program

## Abstract

**Background:**

Medical schools worldwide have employed different practices to facilitate a smooth transition from medical school into the internship phase to promote success in graduates’ future professional life. The College of Medicine at Princess Nourah University (PNU) has developed a unique internship preparatory program focusing on soft and clinical skills.

**Objective:**

The aim of this study was to describe the internship preparatory program (IPP) and evaluate its effectiveness in improving medical students’ transition to internship.

**Materials and methods:**

The IPP for fifth-year medical students at PNU was planned and designed based on students’ needs, the Saudi-Med framework, and similar national/international programs. The one-year longitudinal IPP in 2016–2017 covered four modules conducted as ten workshops focusing on soft skills, clinical skills, and professional development for the future. All data were analyzed by using SPSS version 20.

**Results:**

The IPP was attended and evaluated by 48 participants; 70% of them attended 80% of the IPP workshops. The satisfaction rate for workshop participants was 6.8–8.8 out of 10. Most participants were either satisfied or strongly satisfied with respect to each item on the IPP satisfaction scale; the median satisfaction score was 4 out of 5. A positive significant correlation between the satisfaction score and the number of workshops attended was detected.

**Conclusion:**

The IPP was a satisfying initiative for most participants. It refines their clinical and soft skills, facilitates future planning, and provides a smooth transition from medical school to internship.

## Highlights

• Internship preparatory program (IPP) refines the graduate's clinical skills.
• Internship preparatory program (IPP) refines the graduate's soft skills like communication and collaboration.
• IPP may facilitates future planning for future career.
• IPP provides a smooth transition from medical school to internship.

## Abbreviations

IPP: Internship preparatory program
PNU: Princess Nourah bint Abdulrahman University
Saudi-MED framework: Saudi Medical Education Directives Framework
SMLE: Saudi medical licensure examination
GPA: Grade point average

## 1 Introduction

Throughout the course of medical education, learners face a series of transitions that can be stressful, which can impede their level of preparedness for the new role [1]. This is perhaps justified, given that medical students are often underexposed to acutely unwell patients and to real-life settings during their undergraduate studies [2]. The internship year is considered a critical time for any medical graduate [3], particularly for making career decisions and gaining confidence in clinical skills, communication, and teamwork practices [4]. Many medical schools have tried to ease the transition to internship by offering short periods of shadowing for an intern, known as preinternship placements [5,6].

Most medical school curricula focus on knowledge and clinical skills and overlook essential soft skills. Moreover, in some countries, including Saudi Arabia, traditional internship training is still conducted [7], whereas medical colleges in other countries, are changing or modifying their curricula to embrace new trends in medical education [8]. Recently, a national competence framework has been developed by medical schools in Saudi Arabia to standardized medical training while preserving the autonomy of the college [9].

The College of Medicine at Princess Nourah bint Abdulrahman University (PNU) is one of the newly established medical colleges in the Kingdom of Saudi Arabia and has the unique characteristic of being a female-only medical college in a very prestigious female-only university. The College of Medicine at PNU is competing with more than 36 medical colleges in the government and private sectors around the kingdom. The graduate outcomes and the percentage of their consonance at postgraduate programs reflect the quality of teaching, training, and scholarly activities they received at the undergraduate level. Considering the above, we developed and implemented an internship preparatory program (IPP) in 2016–2017 for the first cohort of fifth-year medical students. The main aim of the program was to facilitate and smooth the transition to the internship phase, help in career planning and the residency matching process, and strengthen the clinical and soft skills required for success in their future professional life.

This study aimed to describe the IPP and evaluate its effectiveness in improving medical students' transition to internship by measuring participants’ satisfaction and to determine whether the IPP improved their clinical and soft skills in the medical field.

## 2 Methods

The study included planning, developing, conducting, and evaluating the IPP. The planning and development phase was conducted to design the IPP according to our students’ needs, the Saudi-Med framework and rigorous literature reviews regarding similar programs nationally and internationally. The needs assessment was conducted in 2016 by surveying our students to determine their views and consider their input in designing the program. The survey focused on the following points to be considered: preferred workshop timing and the selection of topics from the list of workshops that cover soft as well as professional and procedural skills. Additionally, the students were given the opportunity to suggest other related topics to be included in the given list.

Local programs were assessed, especially those conducted at King Saud University and Alfaisal University. However, their focus was on the requirements for residency training programs rather than comprehensive internship preparation. A literature search revealed that communication skills and simulation training approaches are effective and popular with students and may even improve examination and communication performance [10,11]. One of the unique features is that this IPP is one of the few programs that focus on both soft and clinical skills. Thus, the IPP was designed to include the following modules: being an intern, communication skills, professional development, and clinical and procedural skills.

The model on being an intern consisted of two workshops based on the idea of training the students to explore themselves and gauge their needs and competencies to facilitate the transition period from undergraduate to residency life. While preparing this module, we realized that doctors in the internship period must choose their careers, and it is the period in which they are matched with residency options. It is necessary to develop doctors’ choices to be more aware of their own strengths and of any improvement needed. Therefore, in this module, the students were introduced to personality tests; strategic planning; SWOT analysis; career decisions; and an overview of internship rights, duties, clinical rotations, the internship journey, and residency planning. Different training methods were implemented, such as small group discussions, large group interactive sessions, and structured dialog with junior residents who shared their real-life experience with the students.

In our IPP, we especially introduced a training in communication skills and dealing with different communication settings during a medical career as per the Saudi-Med competencies. It is evident in the literature that medical interns are required to communicate with patients and involve them in the provision of health care effectively to ensure that they are treated with respect and dignity. Interns are required to understand and be competent to manage the duties expected from them as team members; recognize the roles and duties of others; and anticipate, recognize, and manage conflict. Our workshop covered the following competencies: maintaining professionalism and patient-doctor relations in different circumstances (e.g., consultation, delivering bad news, complaints), as well as being an efficient team member within the clinical team by communicating ethically and professionally with colleagues, particularly regarding the chain of command, teamwork and the interface with different specialties, and with other healthcare professionals.

The professional development module consisted of three workshops conducted to cover multiple objectives. For example, demonstrating an effective CV writing style, discussing scholarship opportunities and procedures, and attending an orientation on the latest requirements of the Saudi Commission for Health Specialties may facilitate opportunities for matching with national or international postgraduate programs.

Several procedural skills workshops were conducted with a focus on the clinical skills to be performed by medical interns, including ophthalmological and surgical focused on the insertion of Foley's catheter, nasogastric tube, and tube thoracostomy), in addition to basic suture and knot tying skills and airway management skills. Qualified instructors conducted these workshops at the skills and simulation center at PNU.

The program took place at the College of Medicine and Simulation and Skills Development Center either on weekdays after the medical program class or on weekends, with an average of four to eight contact hours weekly. These workshops were prepared and delivered by a group of qualified faculty members as well as external experts in the subject matter. Prereading materials were sent to the students if applicable. After each workshop, trainees were requested to fill out a questionnaire that mainly assessed their feedback about the content and training methods. Further assessment was done in the procedural skills workshops, where every instructor observed and evaluated the performance and needs of each student. These workshops covered common clinical and surgical skills during the internship year, as mentioned above. To assess the performance of each student, a marking checklist was used to determine their level of proficiency in each mentioned skill, and the trainer focused on the skills that needed improvement. Each deficient skill was taught in a station with one student and one trainer.

At the end of the program, the final-year MBBS students were invited to voluntarily participate in this study. They were asked to fill in a self-administered questionnaire online. Almost all students responded, except those who dropped in the final year or did not attend most of the program. The questionnaire clarified the suitability of the student group, and necessary modifications were made. The questionnaire contained multiple aspects, including personal data, academic performance and SMLE scores, residency program interests and acceptance, attendance and assessment of each workshop, overall evaluation of the program, students’ reflection on their internship experiences, and suggestions for program improvement.

One year after IPP completion, participants were contacted to gather information about their grade point average (GPA), Saudi medical licensure examination (SMLE) score, their chosen specialty, their views about the program in relation to their professional life, and their comments and suggestions. The questionnaire used a five-point Likert scale across thirteen domains related to the program organization, the knowledge gained, and skills. The Likert scale scores were as follows: 1, not satisfied at all; 2, not very satisfied; 3, somewhat satisfied; 4, satisfied; and 5, very satisfied. Cronbach's alpha for the questionnaire was 0.928, which reflects excellent internal consistency.

### 2.1 Data analysis

Descriptive analysis of all study variables was conducted in the form of the average and standard deviation or frequency and percentage. Testing the normality distribution of the satisfaction scores was conducted prior to analysis. Satisfaction scores among the studied groups were compared using the *t*-test. Pearson correlation analysis was conducted to estimate the direction and strength of the correlations between variables. A P-value less than 0.05 was considered statistically significant. All data entry and analysis were conducted by SPSS version 20.

### 2.2 Ethical considerations

This research was approved by the Ethical Committee at Princess Nourah bint Abdulrahman University, (IRB number 18–0187). Data were anonymous and used for research purposes.

## 3 Results

The IPP was attended and evaluated by 48 participants, and 35 participants (70%) attended 8 or fewer workshops out of 10. Their GPA scores ranged from 3.05 to 4.81 (average 3.97 ± 0.51). All of them had taken the SMLE, with results ranging from 49 to 90 and an average of 70.0 ± 8.9.

Table 1 shows the number of attendees in individual workshops and their level of satisfaction. Generally, the lowest number of attendees was 26, and the maximum number was 40 (average 35 ± 5.5). Of the maximum satisfaction score of 10, all workshops were rated 6.8 or higher, and 6 workshops were rated 8 or higher.

**Table 1:** The number of participants in individual workshops and their level of satisfaction.

Workshop	Number of attendees	Average satisfaction score
How to write your CV	40	7.5 ± 1.7
Internship: what's next	38	8.4 ± 1.6
Saudi-MED Communication skills	28	7.6 ± 1.6
Internship: out & about	39	8.6 ± 1.4
Scholarship out & about	37	8.6 ± 1.3
Preparation for residency programs	41	8.0 ± 1.5
X-ray reading skills	28	8.8 ± 1.4
Ophthalmoscopy	26	6.8 ± 2.7
Surgical skills	36	8.7 ± 1.4
Airway management skills	37	7.9 ± 2.6

Table 2 shows the individual satisfaction score for each item in the IPP satisfaction survey. Overall, most students were either satisfied (4) or very satisfied (5), in response to each item of the IPP satisfaction scale (median satisfaction score 4 out of 5 in 11 items and 3 in 2 items). The lowest satisfaction was observed in students’ evaluation of the benefits of the IPP in matching for a residency (only 26.7% satisfaction).

**Table 2:** The individual satisfaction score for each item in the IPP satisfaction survey.

Satisfaction items	1 n (%)	2 n (%)	3 n (%)	4 n (%)	5 n (%)	Median score (IQR)
The IPP has addressed my learning needs	1 (2.1)		10 (20.8)	20 (41.7)	17 (35.4)	4 (2)
The timing of the workshops was convenient	1 (2.1)	1 (2.1)	10 (20.8)	18 (37.5)	18 (37.5)	4 (2)
The IPP was well structured			11 (22.9)	17 (35.4)	20 (41.7)	4 (2)
The IPP teaching methods were appropriate to the setting		1 (2.1)	11 (22.9)	16 (33.3)	20 (41.7)	4 (2)
I'm likely to recommend conducting the IPP in the future		1 (2.1)	5 (10.4)	16 (33.3)	26 (54.2)	4.5 (1)
I'm likely to recommend this program to my colleagues		1 (2.1)	6 (12.5)	19 (39.6)	22 (45.8)	4 (1)
The IPP has improved my knowledge about the internship		1 (2.1)	20 (20.8)	22 (45.8)	15 (31.3)	4 (2)
The IPP has improved my procedural skills		1 (2.1)	10 (20.8)	21 (43.8)	16 (33.3)	4 (2)
The IPP has improved my communication skills		1 (2.1)	10 (20.8)	21 (43.8)	16 (33.3)	4 (1)
The IPP has improved my professional development	1 (2.1)	2 (4.2)	13 (27.1)	24 (50.0)	8 (16.7)	4 (1.75)
The IPP assisted my transition from the university to a clinical setting		2 (4.2)	20 (41.7)	15 (31.3)	11 (22.9)	3 (1)
The IPP has improved my performance during the internship	1 (2.1)	2 (4.2)	17 (35.4)	18 (37.5)	10 (20.8)	4 (1)
The IPP assisted in matching with a residency program (optional)	5 (10.4)	9 (22.5)	15 (37.5)	9 (22.5)	2 (4.2)	3 (2)

Students’ level of proficiency in each procedural skill and their satisfaction are charted in Table 3. Their scores for ophthalmological skills were excellent, their scores for airway management skills were good to excellent, and the lowest score was achieved in endotracheal insertion skills (78.7%). Their scores in surgical skills were also good to excellent; however, the lowest proficiency level was found in knot tying skills (54.9%). Of the maximum satisfaction score of 10, all procedural workshops were rated 6.8 and above.

**Table 3:** Students’ level of proficiency in procedural skills and their satisfaction.

Module of clinical & procedural skills		# of attendees	Mean ± SD level of proficiency	Mean % of proficiency	Min.	Max.	Satisfaction score
Ophthalmological skills		26	7.6 ± .9	95.4	4	8	6.8 ± 2.7
Surgical skills	Foley's catheter insertion	36	10.8 ± 2	77.4	5	14	8.7 ± 1.4
	Nasogastric tube insertion	36	15.3 ± 1.8	84.7	10	18	
	Tube thoracostomy	36	12.7 ± 1.4	74.5	10	15	
	Basic suturing skills	36	10.6 ± 1.2	75.4	8	12	
	Knot tying skills	36	4.3 ± 1.8	54.9	2	7	
Airway management skills	Oropharyngeal	37	7.6 ± .8	95.3	6	8	8 ± 2.6
	Nasopharyngeal	37	5.4 ± .8	90.5	3	6	
	Laryngeal	37	11.4 ± 3.6	81.7	4	14	
	Bag and mask	37	8.5 ± 1.7	85.4%	5	10	
	Endotracheal tube insertion	37	20.5 ± 6.6	78.7%	6	26	

## 4 Discussion

We designed and conducted a novel preparatory program to enhance senior medical students’ readiness for an internship. This study aimed to address the feasibility and outcomes of this program. Researchers have found that comprehensive skills workshops may help senior medical students to prepare for their internship and hence for residency training [12]. Moreover, prospective studies were suggested to assess the retention of knowledge and skills acquired by such programs [13,14]. Thus, we asked our participants about the impact of this program on their practice during their internship.

One of the unique features of our program is addressing the needs of the students as well as fulfilling the requirements of national and international residency programs. Two of the most important requirements of a medical student's application for the National Resident Matching Program are the curriculum vitae (CV) and personal statement (PS). The aim of the CV is to give an itemized account of the applicant's accomplishments since the beginning of their undergraduate studies, with the main emphasis on their activities and performance in medical school [15]. In our program, the workshop in CV writing achieved a satisfaction rate of 7.5 ± 1.7. CV writing is an important skill that enables students to present their qualifications and academic and scholarly achievements since in medical school in an attractive way, thus increasing their chances of acceptance and matching with postgraduate programs.

Communication skills training is an essential component of medical school programs [16]. The workshop on communication skills included real-life scenarios and role playing to improve the targeted skills. After completing the workshop, the students used communication skills more frequently while interviewing patients, examining the influence of the illness on the patient's lives, acknowledging the patient's ideas and feelings, and developing empathy. Our results were consistent with a prior study that showed that a short yet intensive small-group workshop for third-year medical students was significantly able to improve specific communication skills from the patient perspective [17]. Additionally, these results support the hypothesis that attitudes and behaviors toward patient-centeredness can be taught during medical studies and can significantly improve students' future clinical performance [[18], [19], [20], [21]].

Our results related to procedural skills workshops demonstrated that the fifth-year students had a good to excellent level of proficiency in each tested skill, except for their unsatisfactory proficiency level in knot tying skills. Formal practical surgical skills training early in the medical curriculum was not found to increase student confidence in practicing basic suture skills during clinical rotations [22]. Brief curricular training may be adequate for some basic skills, e.g., endotracheal insertion, but it is not sufficient in practicing more complex skills, such as surgical suturing [23]. On the other hand, one systematic review showed that boot camps are an effective educational strategy, especially for clinical skills [24]. Multiple studies have also shown the effectiveness of simulation-based learning in improving confidence and preparedness and strengthening medical students' clinical skills before their entrance to clinical wards [[25], [26], [27], [28]].

An instructional teaching method that offered individually tailored and evaluated educational interventions for each set of procedural skills was implemented for the participating students in a formal and safe environment. This strategy improved the students’ performance on basic procedural skills and their satisfaction, which reached a mean of 8.7 out of 10. Using this method, we were able to better prepare the students for hospital practice. Relying solely on the internship year to provide students with procedure skills is inadequate [29].

Although the students’ proficiency level in ophthalmoscopy examination was excellent, their satisfaction score was the lowest in comparison with other procedural workshops. The students reported that the ophthalmoscope technique is easy and that their ophthalmological curricular training was adequate in training them. This workshop did not affect the skills they had already gained, likely because primary medical science education introduces ophthalmoscopy skills to medical students, who are expected to master them during their clinical years [30].

Based on the students’ reflection on their internship experience, our findings support the importance of soft skills such as communication skills, especially delivering bad news, which is a very important skill for any physician. Ferreira da Silva et al. [31] reported that many doctors have not developed sufficient skills in conveying bad news, since the crucial basic points of empathy and a good relationship of trust between doctors and their patients have not been well explored and developed.

In another study that evaluated the skills of postgraduate residents in delivering bad news in a tertiary care hospital, most residents were not satisfied with their skills in delivering bad news and were eager to receive training in this regard [32].

Additionally, the need for preparation for SMLEs and residency programs constitutes a major concern for our students. As we observed from the literature, many programs in different health care institutes are preparing their students to pass the examination. A national survey of U.S. dental hygiene program directors was conducted to determine what strategies their programs employ to prepare students to take the National Board Dental Hygiene Examination, and it showed that most directors (93%) reported they use specific methods and practices to prepare students for the licensing exam [33]. In the United States, medical students use several strategies for United States Medical Licensing Examination (USMLE) Step 1 preparation. At Ohio State University College of Medicine, a yearlong, peer-designed, and peer-led Step 1 review course was conducted for second-year medical students. Course participants had a higher average Step 1 score than nonparticipants (P = .005). Most participants felt that the course was a valuable use of time and would recommend it to future students [34].

To best of our knowledge, this program is one of few national preparatory programs for medical students that cover different aspects of professional skills needed for their transition to internship, hence postgraduate studies. Despite of that, we acknowledge that our research has several limitations, including small sample size, and no control group, since this program was done on the first graduated cohort. Although, using 5-point Likert scale is convenient way to capture attitudes and opinions, however, it is difficult to quantify the difference between satisfied or very satisfied for example. Furthermore, respondents who are confused by the question, or who wish to respond with “not applicable”, which is not available option on the scale itself, they usually respond by giving a midline response (3).

## 5 Conclusion

The study clearly indicates that medical students would transition more smoothly and efficiently if an initiative like our IPP program is included in the final year of study, and that the students appreciated such an initiative. However, specific need analyses and correlation with national/international competency framework (e.g., Saudi-Med) remains a pertinent method to design such a program.

Hands-on training and reflection in the clinical years on communication skills, can improve the intern's competency to handle different patients and situations and can enhance patient centeredness. However, prospective studies are required to ascertain the potential impact of such training program on long term performance. Personalized instructional methods and scaffolding can enhance the students' ability to hone basic procedural skills. National and international residency requirements along with early practice, individually tailored and evaluated educational interventions especially for more complex skills like surgical suturing needs attention.

## Funding

This research did not receive any specific grant from funding agencies in the public, commercial, or not-for-profit sectors.

## Role of the funding source

Not applicable.

## Data availability

Available from the corresponding author upon reasonable request and approval from IRB-PNU.

## Provenance and peer review

Not commissioned externally peer reviewed.

## Declarations of conflict of interest

None.

## Ethical approval

The study was approved by the Institutional Review Board at Princess Nourah bint Abdelrahman University, Riyadh, Saudi Arabia (IRB-PNU: 18–0187).

### Consent

Not Applicable.

## Author contribution

A. A, study concept and design, data collection, manuscript preparation. S.I, study design, data collection and entry, data analysis and interpretation, manuscript preparation. N. A, Corresponding author, study design, manuscript preparation. E. S., study design, manuscript preparation. L. A., study design, data collection and entry, manuscript preparation. A. F., data analysis and interpretation, manuscript preparation. N. M, Study design, manuscript editing. All the authors have read and approved the final version of the manuscript.
